# Functionalized novel carbon dots from bell pepper seeds for sustainable green Edoxaban quantification

**DOI:** 10.1186/s13065-025-01427-z

**Published:** 2025-04-02

**Authors:** Rasha Th. El-Eryan, Mona S. Elshahed, Dalia Mohamed, Azza A. Ashour, Heba T. Elbalkiny

**Affiliations:** 1 Pharmaceutical Analytical Chemistry Department, Faculty of Pharmacy, Helwan University, Cairo, 11795 Egypt; 2 Analytical Chemistry Department, Faculty of Pharmacy, October University for Modern Sciences and Arts (MSA), 6th October City, 11787 Egypt

**Keywords:** Carbon dots, Method’s greenness, BAGI, Complex-GAPI, Eco-scale, Edoxaban

## Abstract

Global warming and the developed worldwide awareness have persuaded efforts to minimize the generated hazardous wastes. As a result, “green” chemical procedures are being gradually included in science for sustainable development. This concept has been extended and inspired chemists to fabricate novel green carbon dots (CDs) from natural plants. Herein, we represent novel CDs synthesized by recycling seeds obtained from bell pepper as fluorescent probe for the determination of Edoxaban tosylate hydrate (EDO) a non-fluorescent drug; we exploit the advantage of the inner filter effect between the absorption peak of the drug and the emission peak of the CDs. This overlap resulted in quenching the fluorescence of CDs by increasing the concentration of EDO within the range 0.80–20.00 µg/mL with a limit of detection 0.23 and 0.22 µg/mL and a limit of quantitation 0.69 and 0.72 µg/mL for Microwave CDs and Plate CDs, respectively, at λ_ex_/λ_em_ 310/409 nm. Two facile preparation techniques for the CDs were used, the microwave-assisted method and the thermal decomposition method, using a single-step approach. The fabricated CDs were characterized using various techniques, including UV-vis, fluorescence and Fourier transform infrared spectroscopies, energy-dispersive X-ray, high-resolution transmission electron microscope, X-ray Diffaction, X-Ray photoelectron spectroscopy and zeta potential. The performance of the synthesized fluorescent probe for the determination of EDO was evaluated according to ICH guidelines. The accomplished results, together with the simplicity, sensitivity, and low cost of the developed probe, recommended its appropriateness for the routine quality control assay of EDO pharmaceutical formulation, as good % recovery was obtained upon the investigation of the marketed tablets with 99.77% and 98.79% recoveries for microwave CDs and plate CDs, respectively. The method’s greenness was evaluated using three integral matrices, the Blue Applicability Grade Index, the Complementary Green Analytical Procedure Index and Analytical Eco-Scale.

**Graphical abstract:**

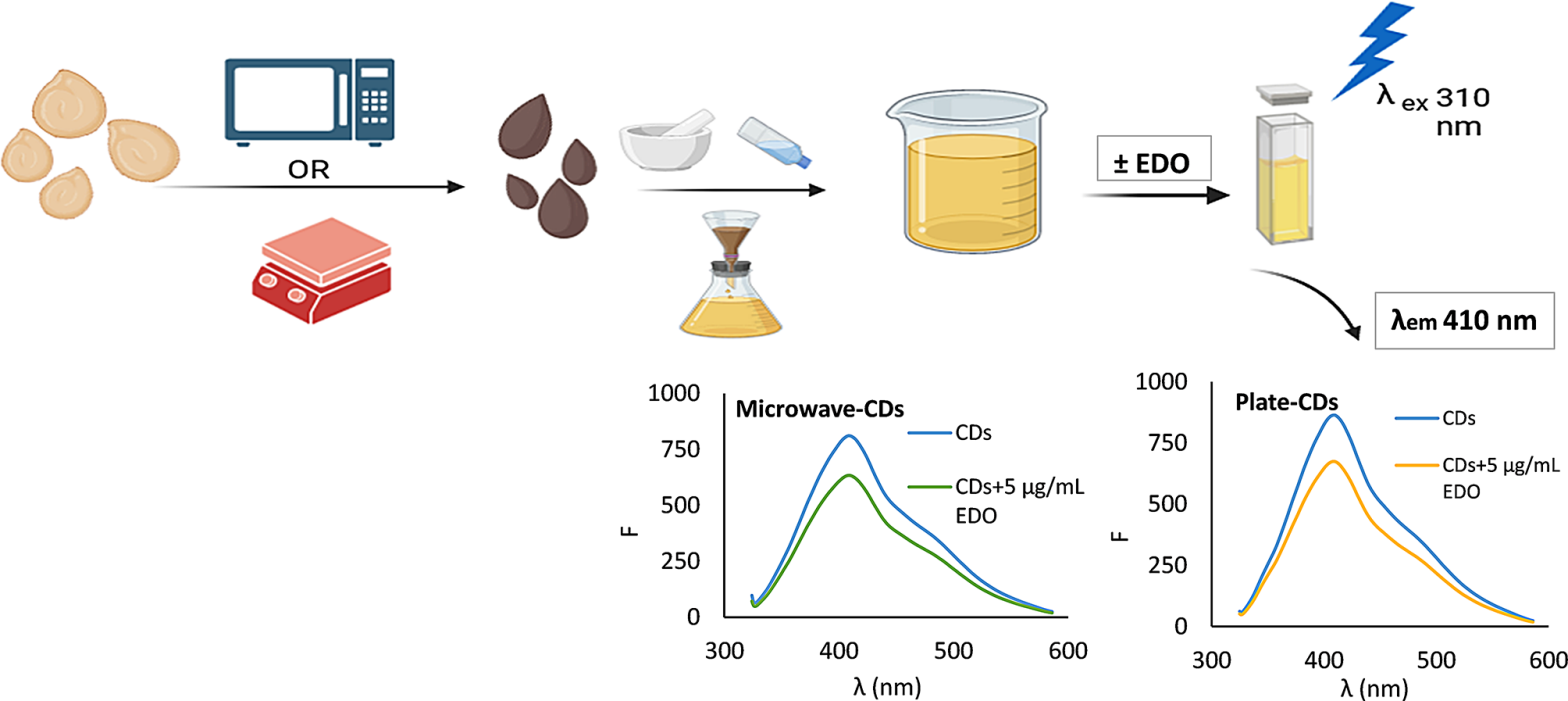

**Supplementary Information:**

The online version contains supplementary material available at 10.1186/s13065-025-01427-z.

## Highlights

- Fabrication of novel carbon dots (CDs) for recycled bell pepper seeds.
- Characterization of the synthesized CDs using various techniques including UV-vis and fluorescence spectroscopies, high-resolution transmission electron microscope, energy-dispersive X-ray, and Fourier transform infrared spectrometer.
- Developing a fluorescent probe for the determination of Edoxaban tosylate hydrate, a non-fluorescent drug, using the synthesized CDs.
- Comparing our results with previously published papers to illustrate the significance of our work.
- Greenness assessment of the synthesis and the analytical methods using three integral matrices, the Blue Applicability Grade Index (BAGI), the Complementary Green Analytical Procedure Index (Complex-GAPI), and The Eco-Scale.

**Supplementary Information:**

The online version contains supplementary material available at 10.1186/s13065-025-01427-z.

## Introduction

Carbon dots (CDs) are zero-dimensional carbonaceous nanospheres with fluorescent properties, their particle size is usually below 10 nm, nonetheless, it may reach up to 60 nm as declared by several reports [1]. Carbon dots have emerged as a promising class of fluorescent materials with diverse applications. These nanoscale carbon-based particles exhibit unique optical properties, including strong fluorescence emission, high photostability, and tunable emission wavelengths. The use of carbon dots as fluorescent probes has gained significant attention in various fields, including sensing where carbon dots have been utilized as sensors for the detection of various analytes [2–4]. The obtained emission spectrum of carbon dots can be modulated in the presence of specific analytes, allowing for sensitive and selective detection [5–10].

For the synthesis of CDs, there are two approaches: top-down and bottom-up. In the first approach, the macroscale carbonaceous material is broken down [11]; while the more favorable approach is the second one, in which the key component is building up single atoms or organic molecules into nanostructures [12, 13]. Thermal decomposition and microwave-assisted methods involve one-step pyrolysis of carbonaceous precursors to form CDs through bottom-up synthesis; the two methods offer quite rapid procedures, and high quantum yield with reasonable cost [14–17].

The inner filter effect (IFE) is one of the common sources of the deterioration of the fluorometric signal. This effect is classified into two types; primary inner filter effect, in which the light is attenuated by the surface solution facing the monochromator so that less intense light fluxes to the subsequent layers of the solution; this effect is usually observed with concentrated solutions [18]. The other type is reported when there is a superposition between the excitation and emission spectrum; it means that emission light released from some particles is reabsorbed again by other unexcited particles in the solution [18]. Fortunately; this effect was employed in the analysis as the superposition between the absorption spectrum of the analyte and the absorption or the emission spectrum of the CDs resulted in quenching the native fluorescence of the nanospheres. By discovering this effect, there is no need for direct interaction between the analytes and the used CDs [19, 20].

Edoxaban tosylate hydrate (EDO), with the chemical structure illustrated in Fig. S1, is a novel selective inhibitor of factor Xa (Factor Xa is responsible for cleaving prothrombin to thrombin) and is used as an oral anticoagulant for prophylaxis in cases of atrial fibrillation or as treatment of pulmonary embolism or venous thromboembolism. EDO is not official in any pharmacopeia yet [21]. To our knowledge, few analytical methods were developed for the determination of EDO including spectrophotometric methods [21, 22], potentiometric [23], chromatographic [24–32], and one spectrofluorometric method based on a derivatization reaction [33]. As the reported spectrofluorometric method involved the consumption of two organic solvents besides the derivatizing chemical reagent and borate buffer, we were motivated to develop a novel, simple, and more green analytical method for the quantitation of EDO.

In the presented work; a new recycled source, bell pepper seeds, was used to prepare CDs by two different and simple procedures, namely the microwave-assisted and the thermal decomposition methods. The obtained CDs were used as fluorescent probe for the determination of the non-fluorescent drug EDO in bulk and pharmaceutical dosage form. Global warming and the developed worldwide awareness have persuaded efforts to minimize the generated hazardous wastes. As a result, “green” chemical procedures are being gradually included in science for sustainable development. This concept is extended and inspired chemists to fabricate novel green CDs from natural plants [34]. Additionally, since the emergence of the term green chemistry in the 1990s, it has become a necessity to consider the impact of the analytical process on the environment [35, 36]. Thus, the greenness of the developed method was evaluated by different green analytical tools; the Analytical Eco-Scale [37], the complementary green analytical procedure index (ComplexGAPI) [38], and the blue applicability grade index (BAGI) [39]. The first tool represents a quantitative scale for the evaluation of greenness. The second tool allows the evaluation of the entire analytical method, starting from the synthesis step, which precedes the analysis step [40]. The BAGI tool allows evaluation of the practicality of the developed analytical method in routine analysis and is used as complementary to the former evaluation [39]. The developed method is considered a simple, sensitive, and good replacement for the tedious, time-consuming, and reagent-consuming HPLC methods specifically for the routine analysis of EDO.

## Experimental

### Materials and reagents

EDO authentic drug (99% purity) was supplied by Rameda Pharmaceutical Company (6th of October City, Egypt). The dosage form Coaguloban^®^ tablets (60 mg Edoxaban per tablet, Marcyrl Pharmaceutical Industries Company, Egypt) and bell pepper were purchased from the local market.

Britton–Robinson (BR) buffer was prepared by mixing 0.4 M phosphoric acid (Sigma-Aldrich, Germany), 0.4 M acetic acid (Sigma-Aldrich, Germany), and 0.4 M boric acid (El-Nasr Company, Cairo, Egypt) in 100-mL volumetric flask to obtain 0.04 M BR buffer from which 10 mL was taken and diluted to 100 mL with double-distilled water. The pH was adjusted using sodium hydroxide (Sigma-Aldrich, Germany) to obtain different buffer solutions in the pH range of 2.0–10.0 [41].

Monobasic potassium phosphate (Sigma-Aldrich, Germany) and sodium hydroxide were used to prepare 0.20 M phosphate buffer at pH 6.0 according to USP pharmacopeia [41].

Quinine sulfate (Aqua Phoenix Scientific, US) was prepared in 0.10 M sulfuric acid (Sigma-Aldrich, Germany) in three different concentrations (20.00, 30.00, and 50.00 µg/mL).

Chloride salts of Na^+^, K^+^, Ca^2+^, Mg^2+^, and Ba^2+^, starch, lactulose, mannitol, and dextrin were obtained from El-Nasr Company, Cairo, Egypt.

### Instrumentation

- The spectrofluorometric measurements were carried out using a JASCO FP-6200 spectrofluorimeter supplied with a xenon flash lamp (150 W), grating monochromator, and a 1-cm quartz cell. Slit widths of 10 nm for both monochromators were used with all measurements except for quantum yield measurements, where 5 nm slit widths were used. Spectra Manager with version 1.54.03 software was used for recording and analyzing the spectra.
- The preparation of CDs was performed using a domestic microwave (700 W, LG), a hot plate (600 W), and a POWER SONIC 410 bench sonicator.
- The pH measurements were carried out using a Hanna pH meter glass electrode (HI 2211 pH).
- Ultraviolet spectrophotometric measurements were performed using a double beam (V-530) JASCO spectrophotometer supplied with matched 1-cm quartz cells.
- The characterization of CDs functional groups was performed using a PerkinElmer Fourier transform infrared spectrometer (FTIR).
- The particle size of CDs was measured from the CDs stock solution utilizing a JEOL JEM-1400 high-resolution transmission electron microscope (HRTEM) with 80 KV accelerating voltage.
- The elemental analysis was performed using the powder obtained after freeze-drying the CDs stock solution utilizing an energy-dispersive X-ray (EDX); JEOL JEM-2300 F.
- For the reference potentiometric method [23]; all the measurements were carried out using a (46-range) Interface digital multimeter connected to the reference electrode (Ag/AgCl/3.0 M KCl) and the fabricated working electrode.

### Preparation of cds

#### Preparation of cds by microwave-assisted method (microwave- cds)

The bell pepper seeds were washed with double-distilled water, and they were added to a glass plate with 5 mL water. The seeds were irradiated in the microwave for 15 min till the color of the seeds was changed to dark brown which indicates the formation of the CDs [16]. For the extraction of the CDs, the charred seeds were grounded in a mortar, nearly 15 mL of double-distilled water was added to the charred powder, the obtained slurry was sonicated for 15 min, and the supernatant was filtrated through a 0.22 μm syringe filter. The obtained yellow clear solution of CDs was stored frozen till its use.

#### Preparation of cds by thermal decomposition using a hot plate (plate- cds)

The washed seeds were placed in a porcelain dish and heated to 280 ⁰C using a hot plate for 40 min till the color of the seeds changed to brown color. The charred seeds were treated in the same way as microwave CDs.

### Stock solution of EDO

Accurately weighed 10.00 mg of EDO was transferred to a 25-mL volumetric flask, and the volume was completed to the mark with double-distilled water. The obtained solution was sonicated for 5 min to ensure complete dissolution of the drug and the solution was marked as a standard stock solution (0.40 mg/mL), which was freshly prepared before its use.

### Preparation of the tablet stock solution

By using Coaguloban^®^ (60 mg Edoxaban per tablet); 10 tablets were crushed in a mortar. A weight equivalent to 10.00 mg EDO was transferred to a 25-mL volumetric flask, and the volume was completed to the mark with double-distilled water. The flask was then sonicated for 15 min. The slurry was filtrated through a 0.45 μm syringe filter. The final obtained filtrate was marked as a tablet stock solution.

### Spectrofluorometric measurements

#### For the construction of the calibration curve

Two groups of 10- mL volumetric flasks were prepared for serial dilution, including one flask as a blank for each group. 300 µL was taken from microwave CDs solution for the first group and 500 µL was taken from the plate CDs solution for the second group using a micropipette. Different accurate volumes from the EDO standard stock solution (covering the linearity range) were added to the volumetric flasks, and the volume was completed to the mark with double-distilled water. The solutions of diluted CDs exhibit a potent fluorescence peak at λ_em_ 409 nm after excitation at λ_ex_ 310 nm which was quantitively attenuated with the addition of the studied drug. The calibration curves were constructed by plotting ΔF (F^o^−F), where F⁰ and F are the fluorescence intensity of CDs without and with EDO, respectively, against the concentration of the drug.

### Quantum yield measurement

The absorbance of specific concentrations of quinine sulfate solution (prepared in 0.1 M sulfuric acid) and CDs solutions were recorded so that those solutions gave absorbance within the range of 0.01–0.1. The same solutions were used to measure the fluorescence peaks and the peak area of the obtained peaks was recorded using a slit width of 5 nm [42].

### Stern Volmer measurement

The Stern Volmer equation was studied to investigate if there is an interaction between EDO and the CDs. The emission spectra for the CDs without and with four different concentrations of the studied drug (0.80, 5.00, 10.00, 15.00 µg/mL) were recorded at λ_ex_/λ_em_ 310/409 at two different temperatures (288 and 318 ⁰K). The ratio between the emission maxima of CDs without and with EDO was plotted against the concentration of EDO [43].

## Results and discussion

The composition analysis of bell pepper seeds showed that the main components are carbohydrates (80.9%), followed by water content (17.27%), fats (11%), proteins (6.3%), and ash (1.81%), besides some elements (K, P, Na, Zn) [44]. For the first time, the bell pepper seeds were recycled and used in the preparation of CDs through a single pyrolysis step. Two methods were used in this work for the preparation of the CDs. By using the microwave-assisted method, water was added to the washed seeds to provide a homogenous nucleation medium and reduce the time required for the carbonization process [45]. In the thermal decomposition method, it was found that the addition of water doesn’t make a difference in the time required for the carbonization process.

### Characterization of carbon Dots

#### Ultraviolet spectrophotometric measurements

**Fig. 1:**
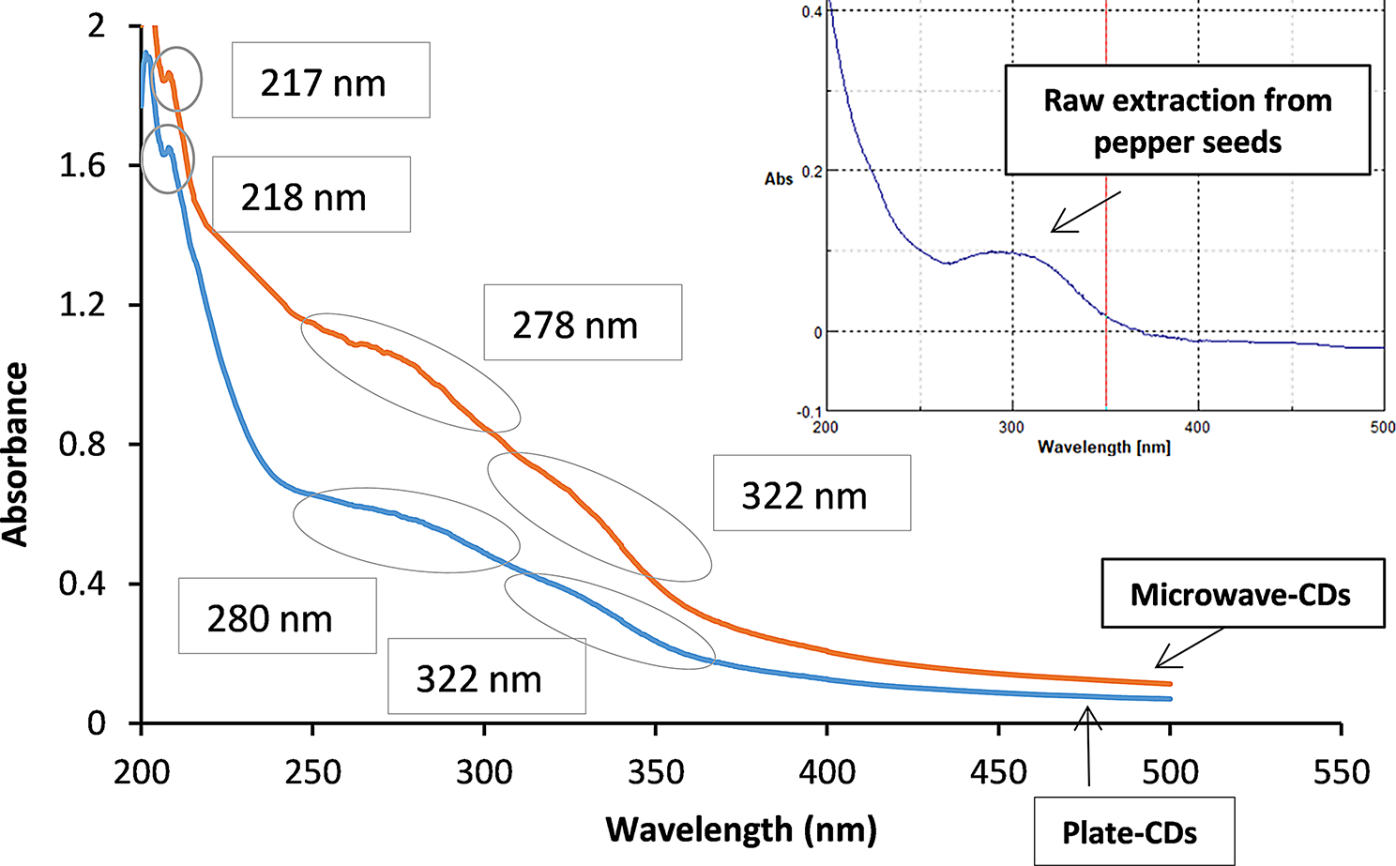
UV absorption spectrum of the synthesized CDs, insert: UV absorption peak of raw extraction from pepper seeds

It was reported that different CDs have different absorption peaks in the UV region, which rely on their structures and also their particle size [46]. Our synthesized CDs showed nearly the same pattern (Fig. 1); the absorption peaks at 217 and 218 nm are attributed to π- π * from C = C, while the broad tail around 280 and 320 nm are attributed to electron transitions n- π * from C-O, C = O bonds and other oxygen surface functional groups of CDs [47]. The characteristic absorption spectrum is accused by restoring a large extent of energy from the excitation wavelengths, which then translated into an intense emission peak [48]. The broadening in the absorption peaks is attributed to the nonperfect uniform sizes of CDs; as different sizes absorb light at slightly different wavelengths [49]. An aqueous extraction from pepper seeds was scanned to obtain its absorption spectrum, which revealed the change between before and after burning the seeds (Fig. 1). The absorption- emission spectrum is illustrated in Fig.S2.

#### Fourier transform infrared spectrometric measurements (FTIR)

**Fig. 2:**
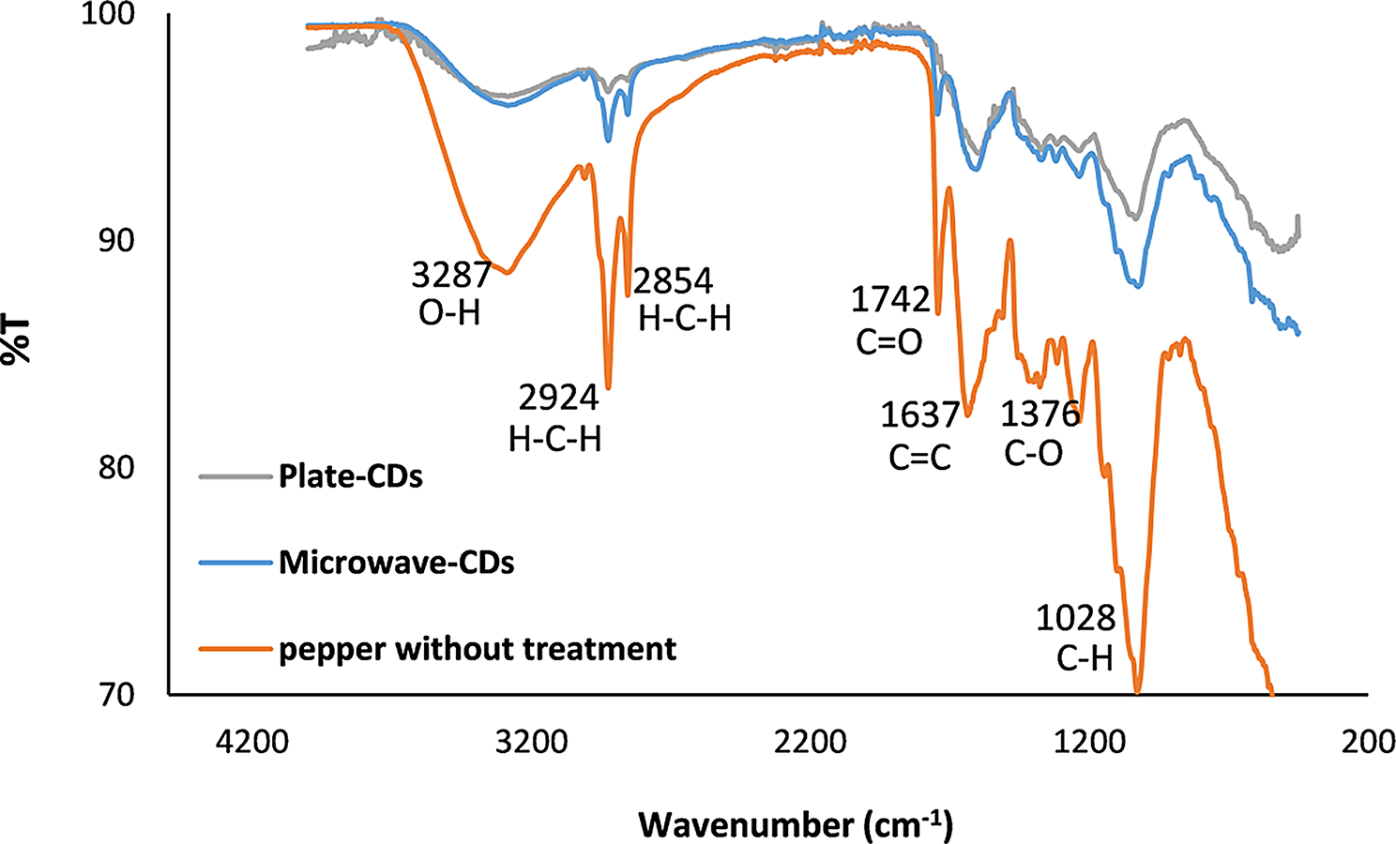
FTIR spectrum for the bell pepper seeds as the precursor and the synthesized CDs

The structure of CDs consists mainly of sp^2^ and sp^3^ carbon atoms with several functional groups attached to their surface [50]. It is a plausible explanation that the synthesized CDs have the same functional groups in the precursor, bell pepper seeds. From Fig. 2, the stretching broad bands in the range of 3287–3283 cm^− 1^ correspond to O-H vibration, while the peaks at 2924 and 2854 cm^− 1^ are attributed to symmetric and asymmetric peaks of CH_2_, the carbonyl group C = O is with a vibrational band in the range 1744–1742 cm^− 1^, C = C has bending vibration in the range of 1604–1596 cm^− 1^ while the vibration band for C-O exists at range 1376–1236 cm^− 1^, the C-O stretching peaks are located in range1315-1024 cm^− 1^ and finally, the C-H bending is located in range 1033–1024 cm^− 1^ [51]. The functional groups of bell pepper seeds indicate that the seeds are composed primarily of hydrocarbons [52]. The FTIR spectrum of the CDs with C = C concluded that the CDs are made of graphite with several functional groups attached to their surface (OH, C = O, C-O, and C-H) [45].

#### High-resolution transmission electron microscope (HRTEM) and X-ray diffaction (XRD)

**Fig. 3:**
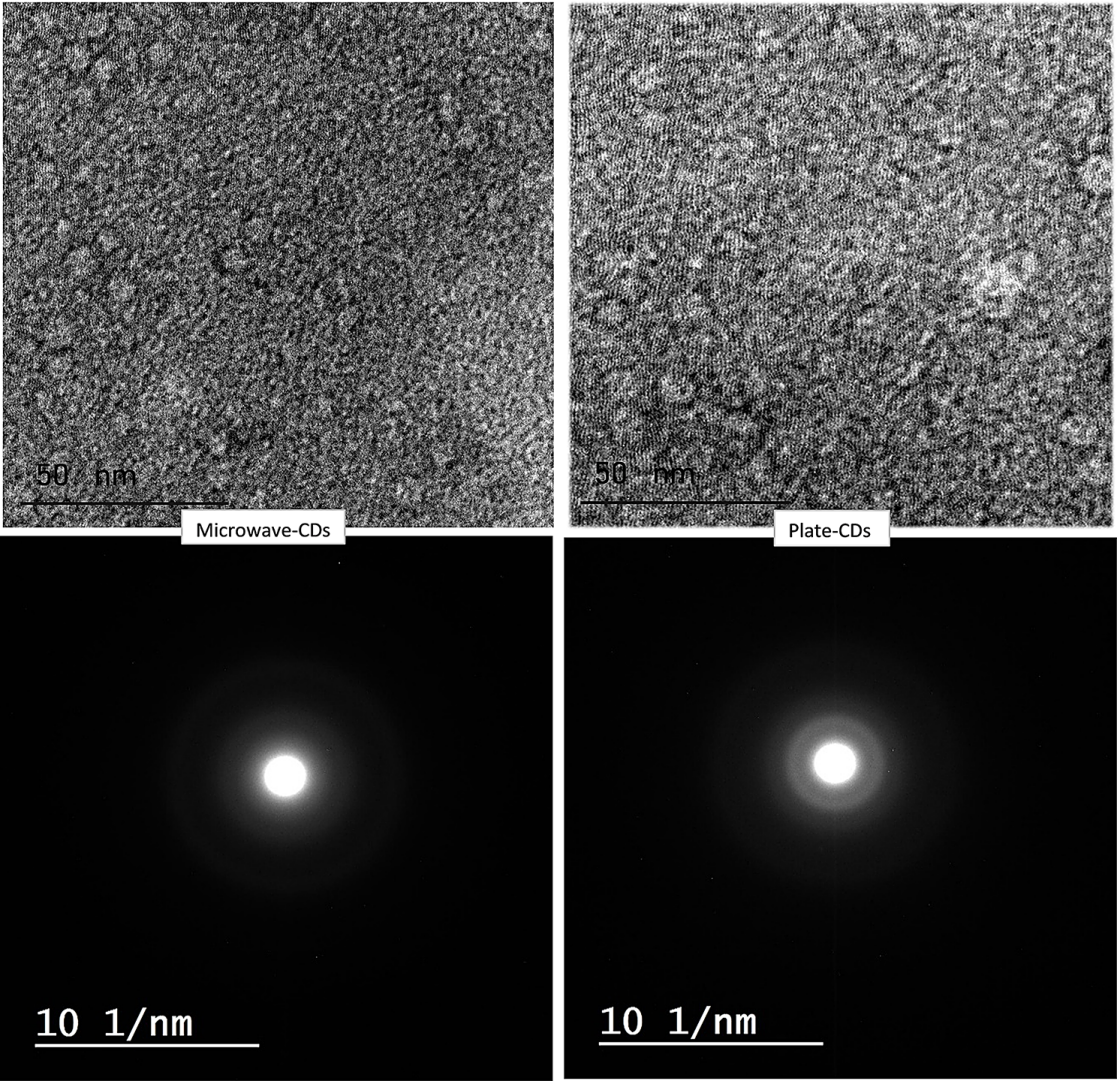
HRTEM for the synthesized CDs

The fabricated carbon dots with the two methods showed particle sizes lower than 3 nm as investigated by HRTEM with narrow size distribution (Fig. 3), it was reported that CDs with particle sizes smaller than 5 nm have significant photoluminescence activity [53]. As shown in Fig. S3. A histogram for particle size distribution revealed that most the particles are wthin the range 1.5–2 nm and 2–2.5 nm for Microwave-CDs and plate CDs, respectively. The normal distribution curves for both nanoparticles revealed that there is narrow distribution of the particle size. From the literature, it was found that the CDs synthesized from natural sources have amorphous shapes, either the whole particles are amorphous or their cores are crystalline and surrounded by an amorphous layer which may also indicate that the precursor is not completely graphitized and serves as a surface group attached to the core of the CDs [45]. The previous explanation complements the HRTEM results obtained from the synthesized CDs, as shown in Fig. 3. The diffuse ring pattern around the spots and the absence of the lattice fringes both indicate that the obtained CDs are amorphous [54, 55]. XRD patterns confirm the disordered carbon structure of the CDs, which is indicated by a wide hump peak extending around 25 degrees [56]. While the low intensity of the peaks is indicative of the small particle size (Fig. S4).

#### Energy-dispersive X-ray (EDX)

The EDX image is useful in determining the elements dispersed on the surface of the CDs with their existing percentage. As illustrated in the provided Fig. S5; the predominant elements are C (49.67% and 49.86% for microwave CDs and plate CDs, respectively) and O (47.77% and 47.47% for microwave CDs and plate CDs, respectively), with trace amounts of residual elements indicating the good purity of the obtained CDs. The elemental analysis results complement those obtained from the FTIR spectrum.

#### X-Ray photoelectron spectroscopy (XPS)

**Fig. 4:**
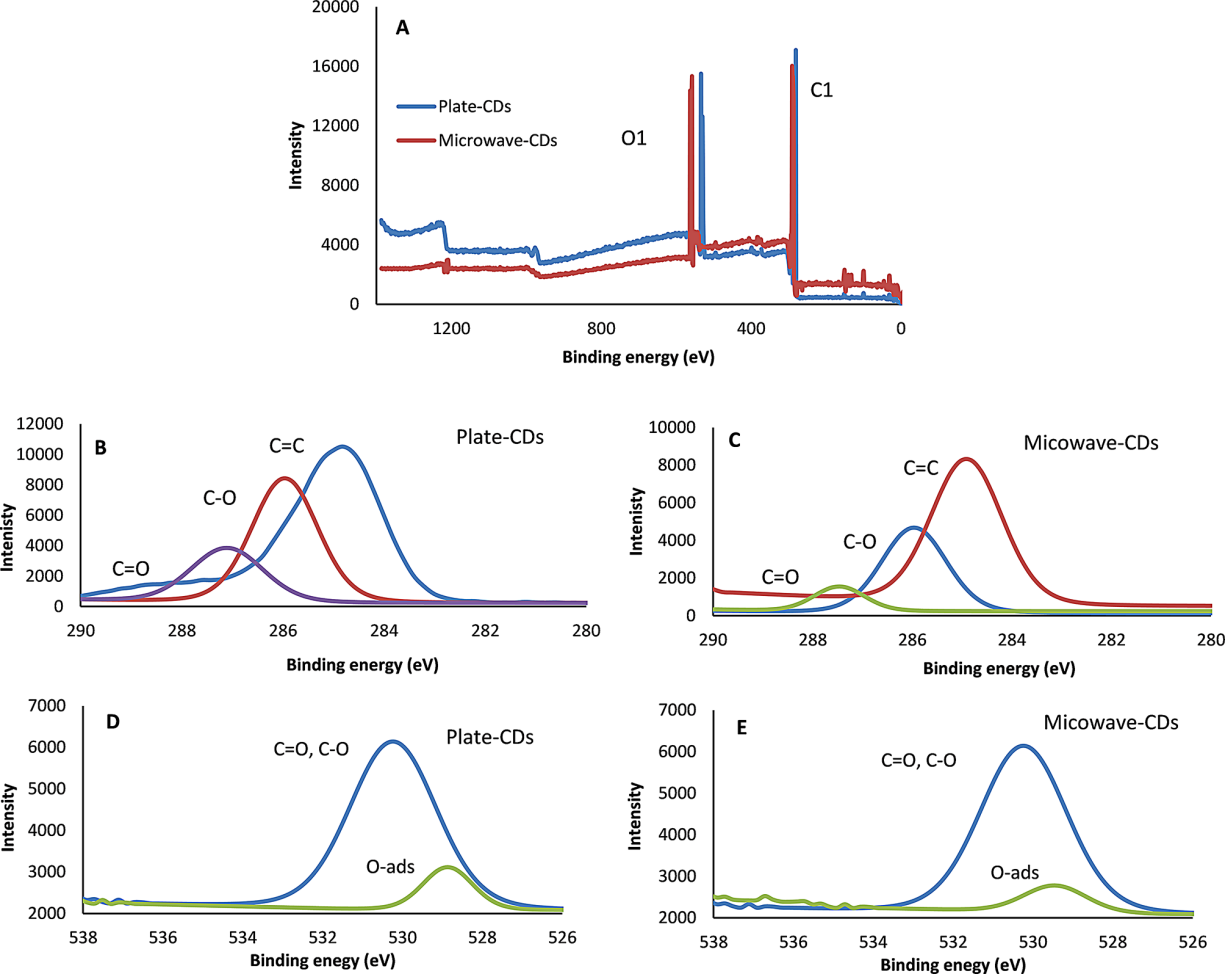
XPS analysis of the synthesized CDs, (**A**)the wide scan, (**B**) and (**c**) are C1 spectra, (**D**) and (**E**) are O1 spectra of plate and microwave CDs, respectively

Based on XPS investigation, the synthesized CDs’ composition comprises carbon (52.32% and 54.85% for plate and microwave CDs, respectively) and oxygen (47.68% and 45.45% for plate and microwave CDs, respectively), as shown in Fig. 4A. Absence of other peaks highlighted the purity of the CDs. The predominant peaks at nearly 285, 286, and 288 eV are corresponding to the graphite carbon, C-O, and C = O, respectively [56] (Fig. 4B and C). Oxygen peaks at nearly 529 and 531 eV are corresponding to physically adsorbed oxygen atoms to the CDs surface and oxygen functional groups [54] (Fig. 4D and E). The obtained results are complementary to those obtained from FTIR.

#### Quantum yield

The quantum yield of carbon dots reflects how efficiently the fluorophore converts the absorbed light into fluorescence. For efficient fluorophores, low concentrations can be used to obtain high fluorescence. The quantum yield was calculated using the following equation [42]:

$$\:Q={Q}_{R}\left[\frac{m}{{m}_{R}}\right]\left[\frac{{n}^{2}}{{n}_{R}^{2}}\right]$$

Where **Q** is the quantum yield of carbon dots, **Q**_**R**_ is the quantum yield of quinine sulfate which is equal to 0.54, and **m** and **m**_**R**_ are the slopes of the regression lines obtained between the integrated fluorescence intensity (which measure the area under the curve, regardless of the wavelength) versus the absorbance for CDs and quinine sulfate, respectively; their values are illustrated in Fig. S6. **n** and **n**_**R**_ are the refractive index for the solvents used for the preparation of CDs (in water) and quinine sulfate (in 0.1 M sulfuric acid), respectively; so this ratio is equal 1. The quantum yield was calculated and was found to be 0.37 and 0.32 for microwave CDs and plate CDs, respectively. The obtained quantum yield values are considered good as the source of the synthesized CDs is a recycled precursor.

#### Zeta potential of the synthesized cds

The value of zeta potential reflects the net value of the electrical charge on the surface of the CDs, the negative sign indicates the presence of functional groups that ionize in the solution and give negatively charged CDs, and vice versa for the positive sign. The value of the zeta potential reflects the stability of the solution; the higher its value, the greater the stability of the solution [57]. In our case, the zeta potentials obtained were − 32 and − 28 for microwave CDs and plate CDs, respectively. The value of the zeta potential reflects the good stability of the system and the presence of negatively ionized functional groups like carboxyl and hydroxyl functional groups. The particle size distribution was measured using a particle size analyzer, which revealed polydispersity values (PdI) 0.34 and 0.39 for microwave CDs and plate CDs, respectively, indicating the narrow distribution of the particle size [58].

### Optimization of experimental condition

#### Effect of excitation wavelength

**Fig. 5:**
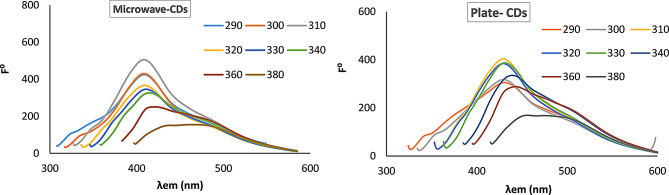
Effect of different excitation wavelength (290, 300, 310, 320, 330, 340, 360 and 380 nm) on the emission spectrum of the synthesized CDs

The synthesized CDs were irradiated with different excitation wavelengths to investigate the effect of excitation wavelength on the emission spectrum (Fig. 5). It was found that the maximum emission spectrum at λ_em_ 409 nm was obtained by utilizing λ_ex_ at 310 nm for both types of CDs. A slight shift in λ_em_ was observed after excitation at 360 nm for microwave CDs and 340 nm for plate CDs. The independence of the emission peak on the excitation wavelength is attributed to the uniform particle size distribution [48]. The shoulder peaks may be attributed to the particle size distribution; smaller particles tend to emit at shorter wavelengths. Also, the localized surface functional groups introduce localized energy levels that contribute to shoulder peaks [59].

#### Effect of pH

The protonation and deprotonation of the functional groups on the surface of CDs impress its emission spectrum [60]. Also, the pH may affect the absorption peak of the studied drug.

**Fig. 6:**
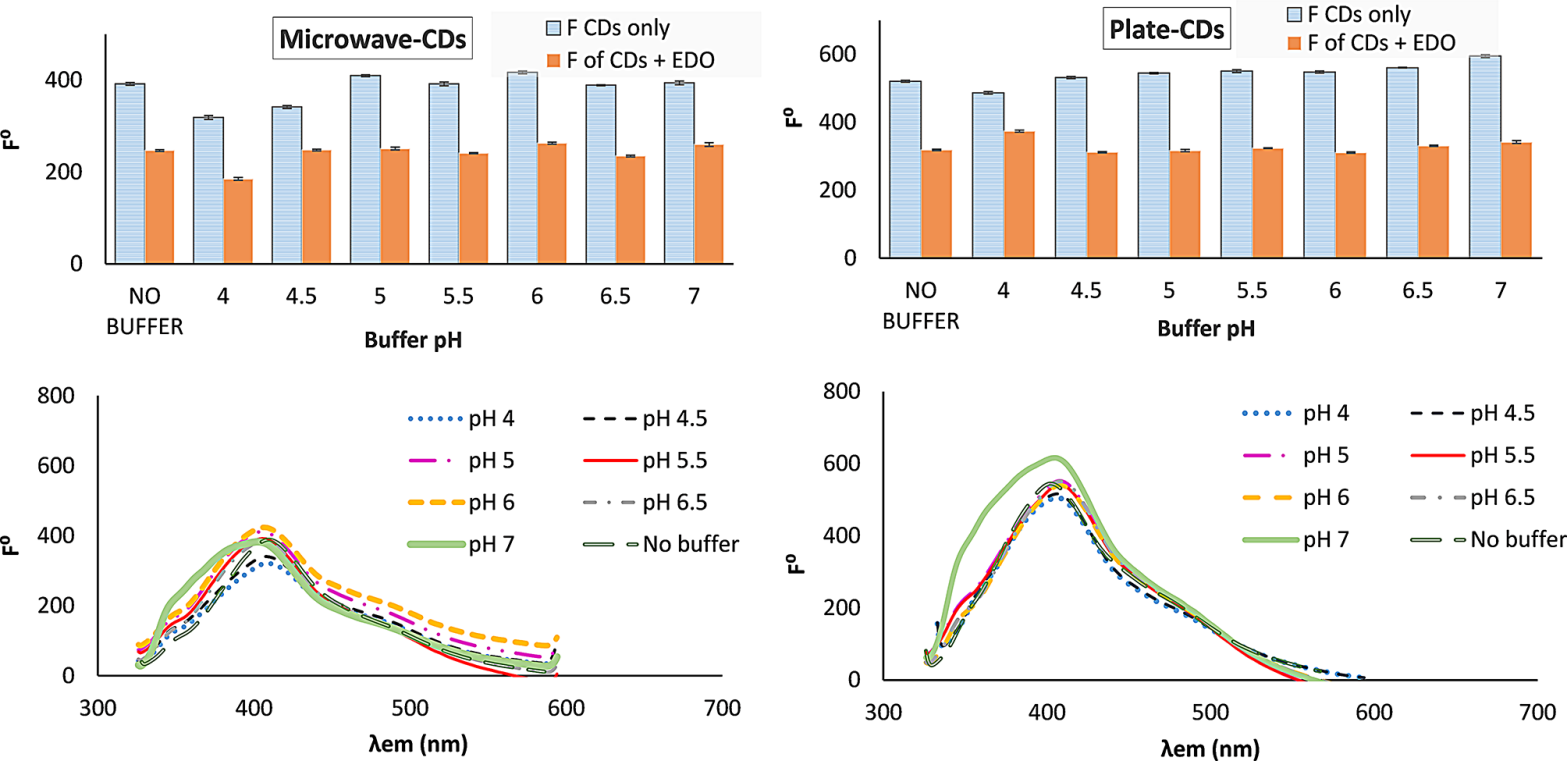
Effect of pH on the synthesized CDs and on the quenching mechanism using 1 mL BR buffer at different pH, at λ_ex_/λ_em_ 310/409 nm, the spectrum is corresponding to CDs with EDO

The effect of pH change was studied on the emission spectrum of CDs and the quenching mechanism; using BR buffer over the pH range 2–10. For the emission spectrum (Fig. 6); at pH lower than 4 no emission peak was observed. The fluorescence intensity was increased as the pH was increased from pH 4 then there was a plateau at pH 5-6.5 and 4.5–6.5 for microwave- CDs and plate- CDs, respectively. At pH above 7, there was deterioration in the peak shape for both CDs. There was a slight shift in λ_em_ from 409 to 402 for microwave CDs and from 408 to 405 for plate CDs as the pH increased from 2 to 8. The addition of buffer solutions with different pH doesn’t reflect an enhancement in the quenching intensity as compared to the absence of the buffer **(**Fig. 6). Further investigations were performed using phosphate buffer at pH 6.0, and it was found that there is a quenching effect on the fluorescence of CDs. We concluded from this study that the pH of the drug solution and tablet solution with the CDs (without the addition of buffer) were within the stable pH range, so there is no need to use a buffer solution. On the other side, we gain an enhancement in the greenness of the method.

#### Effect of incubation time

The drug was added to the prepared CDs and the effect of incubation time was studied over the range of 1 to 60 min. It was observed that there is no change in the ΔF after the addition of EDO over the specified time, indicating that the fluorescence of CDs is quenched quickly upon the addition of the studied drug, which also indicates that the quenching mechanism is attributed to IFE.

### Mechanism of fluorescence quenching

The overlap between the absorption spectrum of EDO and the emission spectrum of CDs verifies that the attenuation in the native fluorescence of CDs upon the addition of EDO is attributed to the IFE. Quenching the fluorescence of CDs can also be attributed to a complementary mechanism due to the interaction between the studied analyte and the CDs through static or dynamic mechanisms. The Stern Volmer equation was applied at two different temperatures to investigate this effect [42]:

$$\:{F}^{0}/F\hspace{0.17em}=\hspace{0.17em}1\hspace{0.17em}+{K}_{sv}\hspace{0.17em}\:\left[Q\right]$$

Where **F⁰/F** is the ratio between the emission maxima of CDs without and with EDO at λ_ex_/λ_em_ 310/409 nm, respectively. **K**_**sv**_ is the Stern Volmer constant, and **[Q]** is the analyte concentration (EDO).

Upon increasing the temperature, **K**_**sv**_ decreases if it is static quenching, while its value increases in the case of dynamic quenching. In our study, as shown in Fig. S7, the **K**_**sv**_ value remained constant at the two different temperatures (0.0305 and 0.0294 for microwave CDs and 0.0549 and 0.0593 for plate CDs at 318 and 288 °K, respectively); these results prove that there is no interaction between the studied drug and the prepared CDs [61].

### Method validation

**Fig. 7:**
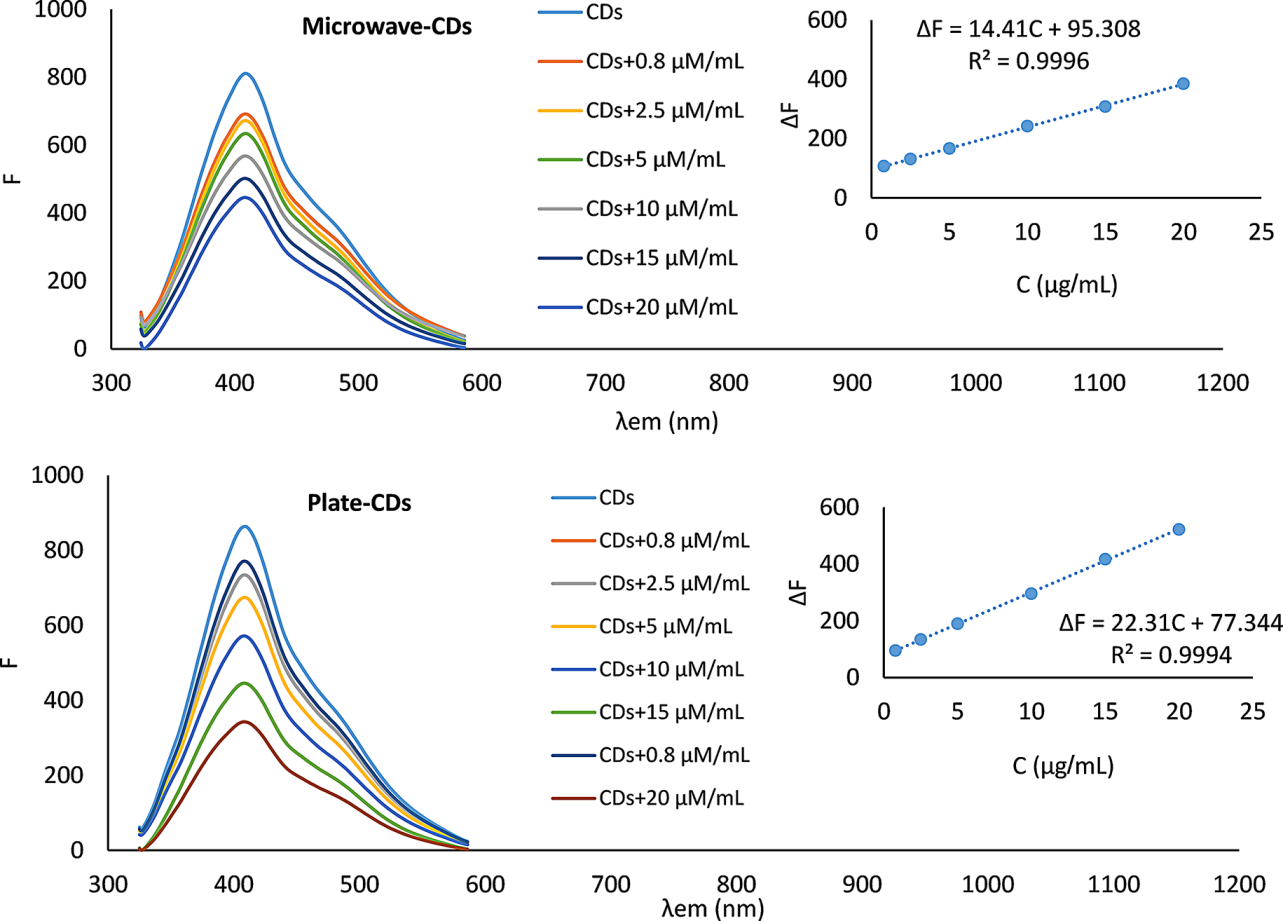
The quenching response of the synthesized CDs probe toward different concentrations of EDO at λ_ex_/λ_em_ 310/409 nm, Inserts: are the corresponding calibration curves

The proposed analytical method was validated in compliance with ICH guidelines [62] concerning linearity and range, accuracy, precision, and robustness; the results are shown in Table 1. The proposed method showed good linearity over the concentration range of 0.80–20.00 µg/mL (Fig. 7). The accuracy and precision were studied using three different concentrations and three replicates. The two synthesized CDs showed acceptable accuracy and precision (Table 1).

In order to investigate the possible interference effect, different possible excipients used in the preparation of the tablets were investigated, like starch, lactulose, mannitol, and dextrin. Each possible interferant was added to the prepared CDs, and the FI was measured with and without the addition of the interferent (Fig. S8). Also, the method’s selectivity was investigated using different metal ions like Na^+^, K^+^, Ca^2+^, Mg^2+^, and Ba^2+^. Each element was added to the prepared CDs, and the FI was measured, then the FI was measured in the presence of the metal ions and the studied drug (Fig. S8). As illustrated in the figure, the results showed that no interference from those exciepents was found and good selectivity toward the studied drug was obtained in the presence of the metal ions.

**Table 1:** Regression parameters and validation sheet for determination of EDO standard solution by the proposed method

Parameters	Microwave CDs	Plate CDs
Excitation wavelength	310 nm	310 nm
Emission Wavelength	409 nm	409 nm
Linearity range	0.80–20.00 µg/mL	0.80–20.00 µg/mL
Slope	14.41	22.31
Coefficient of determination (R2)	0.9996	0.9994
Standard deviation of the residuals (S_y/x_)	2.30	3.84
Limit of detection (LOD) ^a^	0.23 µg/mL	0.22 µg/mL
Limit of quantitation (LOQ) ^b^	0.69 µg/mL	0.72 µg/mL
Mean %recovery ^c^ ± SD	100.11 ± 1.35	100.05 ± 1.61
Intra-day precision ^d^	100.55 ± 0.86	98.55 ± 0.98
Inter-day precision ^e^	101.31 ± 0.59	100.35 ± 1.15

^a^ LOD = 3.3σ/ S (σ is the standard deviation of the response and S the slope of the calibration curve)

^b^ LOQ = 10σ/ S (σ is the standard deviation of the response and S the slope of the calibration curve)

^c^ Average recoveries of six different concentrations

^d^ Average recoveries of three replicates for three different concentrations (0.80, 10.00, and 20.00 µg/mL), on the same day

^e^ Average recoveries of three replicates for three different concentrations (0.80, 10.00, and 20.00 µg/mL), on different days

### Application

#### Analysis of the drug in the tablet dosage form

The proposed method was successfully applied to determine EDO content in marketed tablets. The results stated in Table 2 revealed that the proposed method provides comparable results with the declared amount in the tablets and proves that the proposed method can be used for routine analysis in quality control laboratories. The standard addition method was also performed to exclude any effect from the matrices; satisfying results were obtained as shown in Table 2, indicating the reliability of our proposed method for analysis of the studied drug in its tablet dosage form. The obtained results were statistically compared with those obtained from a potentiometric reference method utilizing a screen-printed electrode modified with EDO-Phosphotungestic acid as an ion pair and dibutyl phthalate [23]; good accuracy and precision were obtained as indicated by the student *t*-test and the F- value, respectively (Table 3).

**Table 2:** Quantitative determination of EDO in Coaguloban^®^ tablets by the proposed method using direct and standard addition methods

Direct method												
Coaguloban^®^ (60 mg Edoxaban per tablet)	Parameters			Micro-CDs					Plate-CD			
	Conc. taken (µg/mL)			0.8		5		20	0.8	5		20
intra-day precision	Mean Recovery %*			99.67		98.10		101.55	98.38	100.11		98.77
	RSD%			1.21		0.97		1.04	0.95	1.07		1.13
Inter-day precision	Mean Recovery %*			97.94		100.88		99.81	97.82	101.01		97.43
	RSD%			1.31		1.12		2.10	1.97	1.27		0.98
**Standard addition method**												
**Parameters**		**Conc. taken** **(µg/ mL)**	**Conc. added** **(µg/mL)**		**Micro-CDs**				**Plate CDs**			
					**Conc. found** **(µg/ mL)**		**%Recovery**		**Conc. found** **(µg/ mL)**		**%Recovery**	
Coaguloban^®^ (60 mg Edoxaban per tablet)		5	2.5		7.61		101.47		7.65		102.00	
			5		9.97		99.70		9.89		98.90	
			10		14.83		98.87		15.05		100.33	
% Mean Recovery*							100.01				100.41	
% RSD							1.33				1.55	

*Each result is an average of three replications

**Table 3:** Statistical comparison between the proposed method and the potentiometric reference method

Parameters	Micro-CDs	Plate-CD	Reference method ^b^
Mean Recovery % ^a^	99.77	98.79	99.58
RSD%	1.73	0.42	0.77
N	3	3	3
Student *t*-test ^c^	0.77 (2.78)	2.25 (2.78)	
F-test ^c^	5.34 (19)	1.50 (19)	

^a^ Each result is an average of three determinations. ^b^ Potentiometric method utilizing screen-printed electrode modified with EDO-Phosphotungestic acid as an ion pair and dibutyl phthalate [23]. ^c^ the tabulate t and F values at *p* = 0.05 [63]

### Assessment of the method’s greenness

#### Blue applicability grade index (BAGI)

**Fig. 8:**
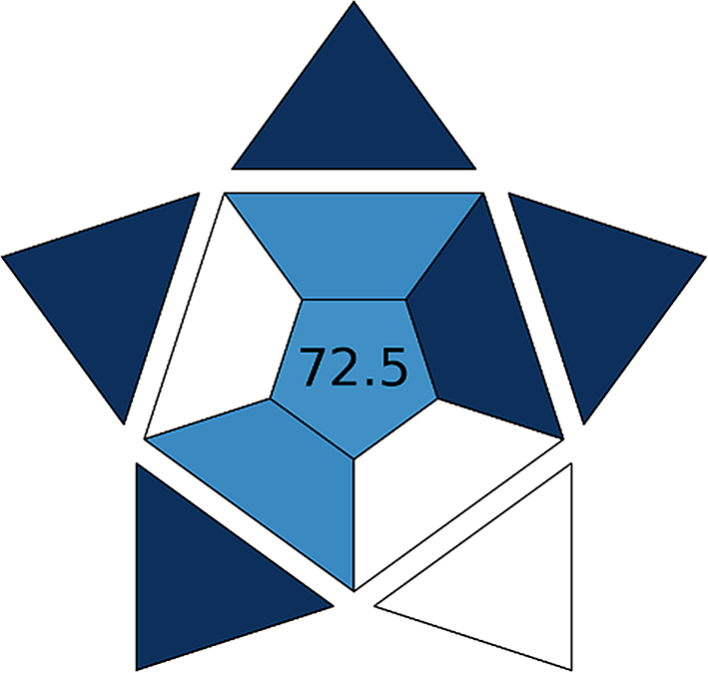
BAGI pictogram for our developed analytical method

It is the most recently developed matrix for the evaluation of the method’s greenness [39]. The metric is represented with a pictogram with a dark blue color indicating high applicability, a blue color reflecting medium applicability, a light blue color for low applicability, and finally a white color indicating no applicability. The number at the center is a mirror for the overall score for the analytical method, a score of 100 means excellent applicability. The method is considered practical when the overall score is more than 60. For our developed method (Fig. 8), it is considered a practical method with a score of 72. The white regions in the pictogram correspond to the number of analytes that can be simultaneously prepared and analyzed; as the drug is available alone in its marketed form, it was analyzed alone in the developed analytical method.

#### Complementary green analytical procedure index (ComplexGAPI)

**Fig. 9:**
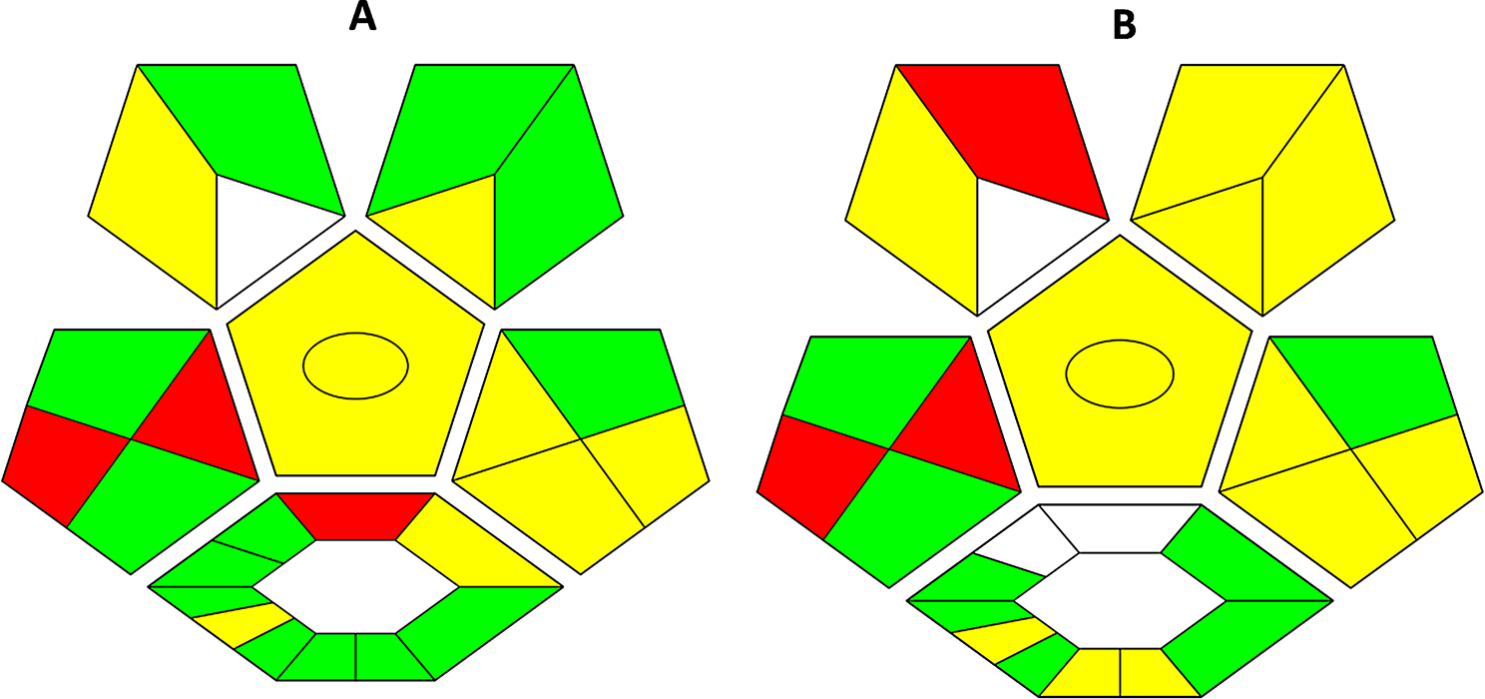
ComplexGAPI assessment for our developed analytical method (**A**) and previously developed spectrofluorometric method (**B**)

This tool is one of the most established tools for the evaluation of the method’s greenness. The glyph is represented by a color scale; the green color indicates a low hazard in the environment, the yellow color reflects a medium hazard, and the red color is an alert for a hazardous step [38]. As shown in Fig. 9; two red alerts in the first pictogram correspond to the transport of the sample and the off-line collection of it, which are mandatory steps in pharmaceutical companies where the production and quality control departments are separated. The red region in the hexagonal shape reflects the low %yield of the synthesized CDs and the high waste, but fortunately, the waste material is a natural source (bell pepper seeds), which is not hazardous to the environment. Using the hot plate instead of the microwave in the synthesis of the CDs resulted in more than 3 times the consumption in energy, but it is still less than 1.5 kWh; so both instrumentations were colored yellow, and there is no difference between them in the ComplexGAPI assessment. In comparison with the previously established spectrofluorometric method [33], our method showed better greenness as the former method involved a derivatization step, using two organic solvents besides the derivatizing reagent and borate buffer.

#### Analytical Eco-Scale

This metric has the advantage of considering all the chemical reagents and solvents used throughout the analytical method; not only the most hazardous ones as described in other metrics [37]. The green tool depends on calculating the penalty points and subtracting the total from 100; the obtained result is the Eco-Scale of the method. For our developed method, the calculated Eco-Scale was found to be 94 which reflects excellent green analysis. By calculating the same score for the reported spectrofluorometric method [33], it was found to be 73, which represents an acceptable green analysis. The results are shown in Table 4.

**Table 4:** Analytical Eco-Scale for our developed analytical method and previously developed spectrofluorometric method

Our developed method		Previously developed spectrofluorometric method	
**Reagents**			
Recycled bell pepper seeds	0	9-fluorenyl methyl chloroformate	4
		Acetonitrile	6
		Boric acid	2
		Sodium chloride	2
		Methanol	8
**Instrument**			
spectrofluorimetry	0	spectrofluorimetry	0
pH meter	0	pH meter	1
Sonicator	1	Sonicator	1
Microwave/ hot plate	1		
Occupational hazard	0	Occupational hazard	0
Waste	4	Waste	4
**Total penalty points**	**6**	**Total penalty points**	**27**
**Analytical Eco-Scale**	**94**	**Analytical Eco-Scale**	**73**

### Comparison with previously developed spectroscopic work for analysis of EDO

Herein is a comparison between the developed method and the previously established spectroscopic methods. Our method introduces a green analytical method with a reasonable linearity range as shown in Table 5.

**Table 5:** Comparison between the developed method and the previously established spectroscopic methods

Technique	Principle	Concentration range	Application	Reference
Spectrophotometry	Measuring the absorbance of the drug at λmax at 291.2 nm. The stock solution was prepared in methanol.	2.00–10.00 µg/mL	Tablets	[21]
	Measuring the absorbance of the drug at 289 nm, The drug stock solution was prepared in methanol.	5.00–25.00 µg/mL	Synthetic tablet	[22]
Spectrofluorimetry	Derivitization reaction between the amino group in the studied drug and the chloride atom in 9-fluorenyl methyl chloroformate in the presence of borate buffer (pH 9). The reaction took 15 min, and the fluorescence of the formed product was measured after it was dissolved in a sufficient amount of acetonitrile. The drug solution was prepared in a methanol/water mixture.	5.00–50.00 ng/mL	Tablets	[33]
	Measuring the quenching in the fluorescence when the studied drug was added to an aqueous solution of newly synthesized CDs. The CDs are prepared from natural source using a facile procedure. The stock solution was prepared in water.	0.80–20.00 µg/mL	Tablets	Our method

## Conclusion

Novel carbon dots were fabricated by recycling the bell pepper seeds. Two simple carbonization techniques were used, microwave-assisted and thermal decomposition, where the obtained results confirmed that there is no difference in the performance of both CDs. The synthesized CDs showed good properties such as water solubility, environment stability (pH), and potent fluorescence activity besides the facile and low-cost production process. The CDs were used for the determination of the non-fluorescent drug, Edoxaban tosylate hydrate, with good sensitivity, and the results revealed that the probe can be successfully used in the analysis of pharmaceutical samples without complicated pre-treatment steps. The method’s greenness was evaluated by three complementary metrics; BAGI, Complex-GAPI, and Analytical Eco-Scale, where the obtained profiles proved the environmentally friendly nature of the proposed method.

## Electronic supplementary material

Below is the link to the electronic supplementary material.

Supplementary Material 1

## Data Availability

No datasets were generated or analysed during the current study.

## Abbreviations

CDs: Carbon dots
EDO: Edoxaban tosylate monohtdate
IFE: Inner filter effect
ComplexGAPI: Complementary green analytical procedure index
BAGI: Blue applicability grade index
BR buffer: Britton-Robinson
