# Supplementary material for: Functionalized novel carbon dots from bell pepper seeds for sustainable green Edoxaban quantification
Source: BMC Chem. 2025 Apr 2;19(1):85. doi: 10.1186/s13065-025-01427-z (PMC11966911; doi:10.1186/s13065-025-01427-z)
Supplement: Supplementary file 1 — Supplementary Material 1 [file 13065_2025_1427_MOESM1_ESM.docx]

**Functionalized novel carbon dots from bell pepper seeds for sustainable green Edoxaban quantification**

Rasha Th. El-Eryan ^a^, Mona S. Elshahed ^a^, Dalia Mohamed^a^, Azza A. Ashour ^a*^, Heba T. Elbalkiny^b^

^a^ Pharmaceutical Analytical Chemistry Department, Faculty of Pharmacy, Helwan University, 11795, Cairo, Egypt

^b^ Analytical Chemistry Department, Faculty of Pharmacy, October University for Modern Sciences and Arts (MSA), 11787 6th October City, Egypt

^*^Corresponding Author E-mail Address [azza.abdellateef@pharm.helwan.edu.eg]

**Fig. S1.** Chemical structure of EDO.

**Fig. S2.** Absorption-emission spectrum for the synthesized CDs.

**Fig. S3.** Histograms and normal size distribution curves for both synthesized CDs

**Fig. S4.** XRD pattern of the synthesized CDs.

**Fig. S5.** EDX spectra of the synthesized CDs. Inserts: the elemental analysis of the CDs

**Fig. S6.** Plot of the integrated fluorescence intensity against the absorbance for quinine sulfate and the two synthesized CDs

**Fig. S7.** Stern Volmer plots for the synthesized CDs at 318 and 288 ⁰K

**Fig. S8.** The selectivity study: response of the introduced CDs toward different possible interferant. The selectivity study: response of the introduced CDs toward the metal ions without and with presence of EDO.


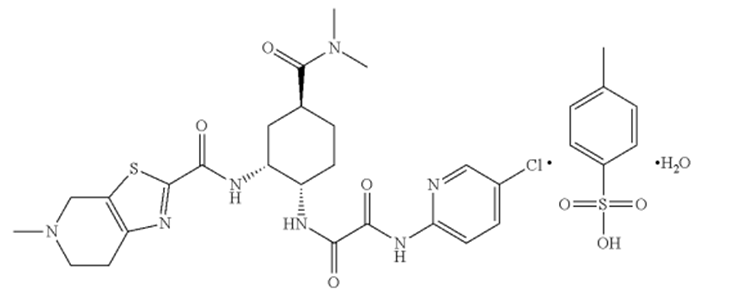


**Fig. S1**

**
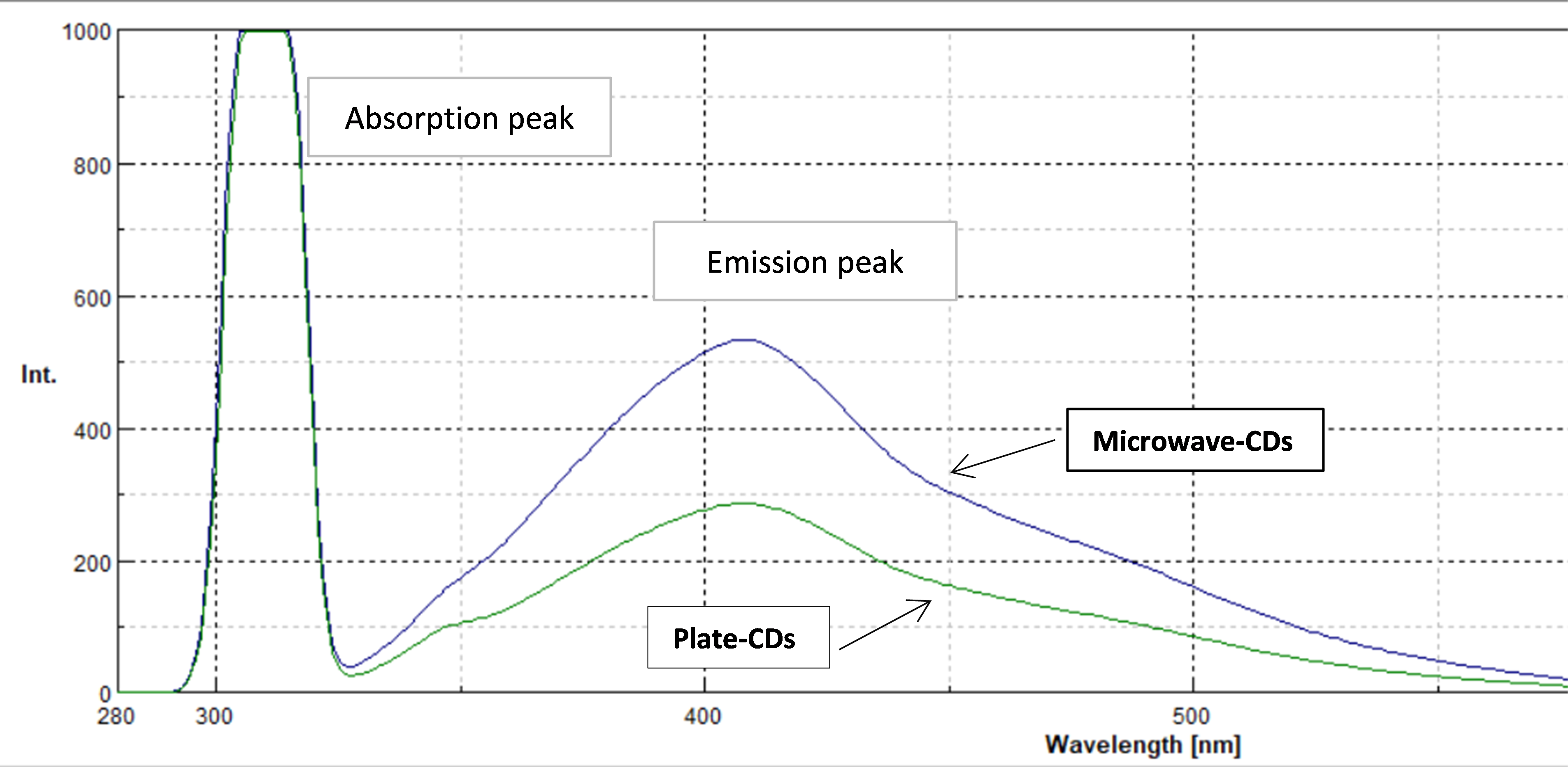
**

**Fig. S2**

**
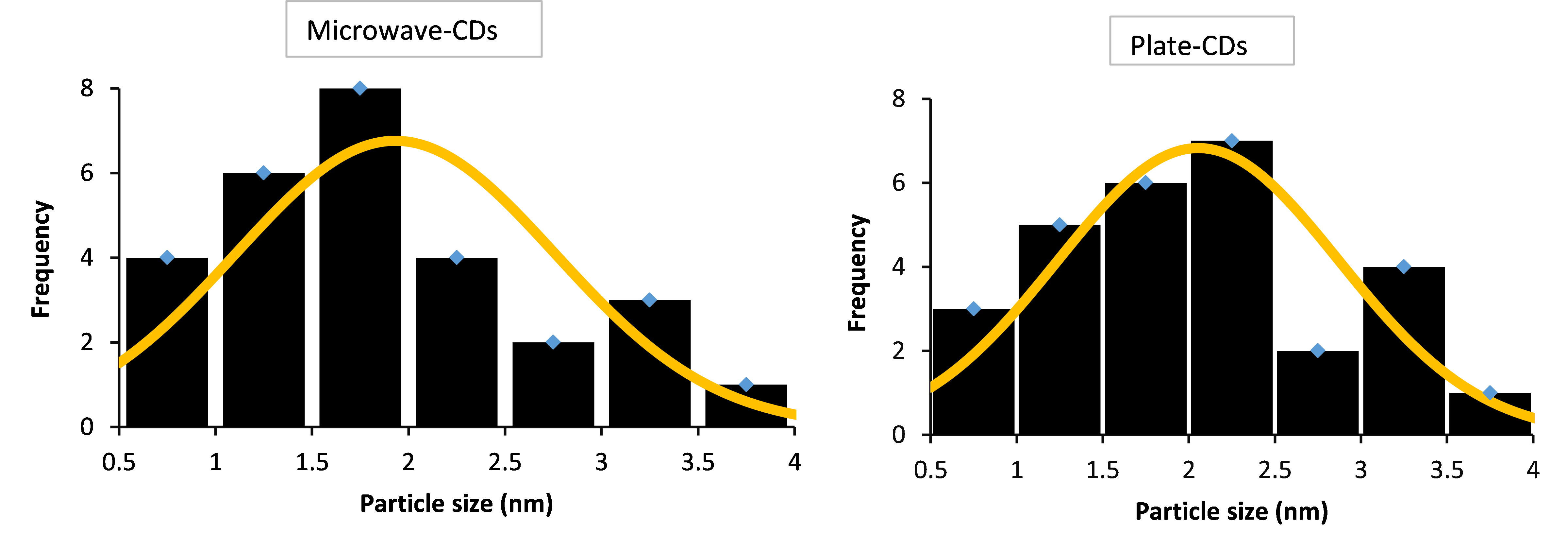
**

**Fig. S3**


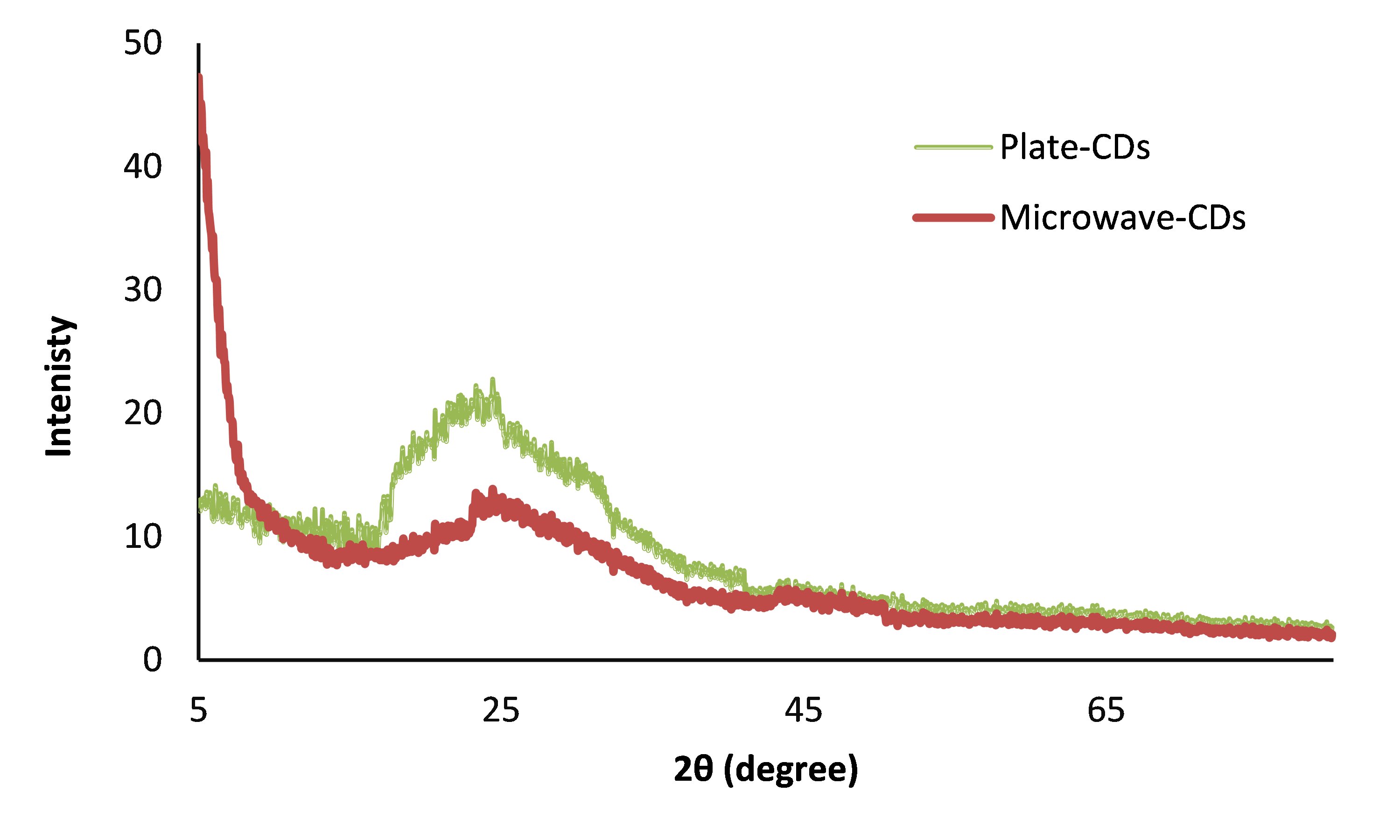


**Fig. S4**


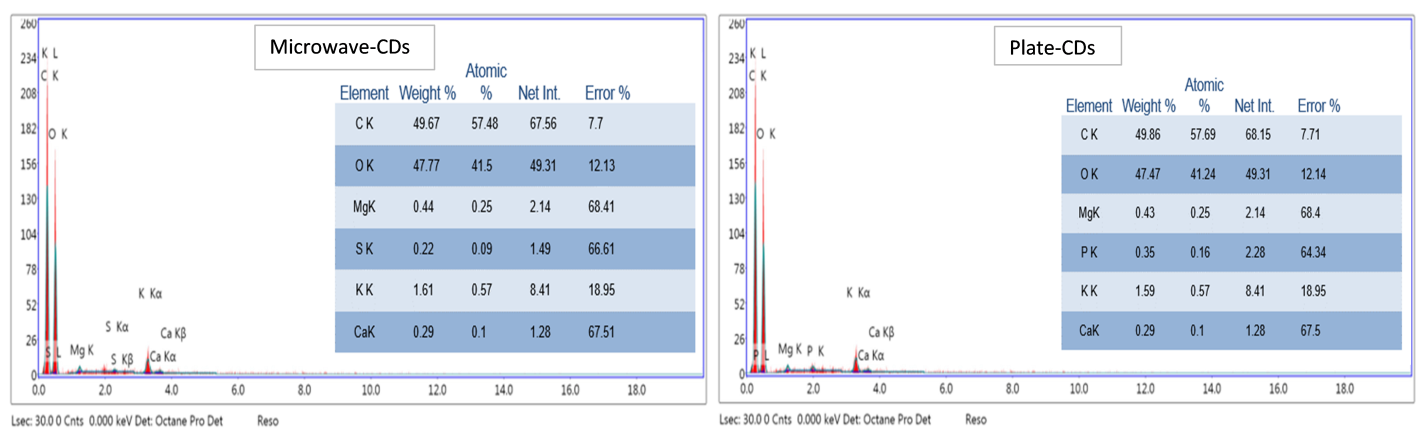


**Fig. S5**


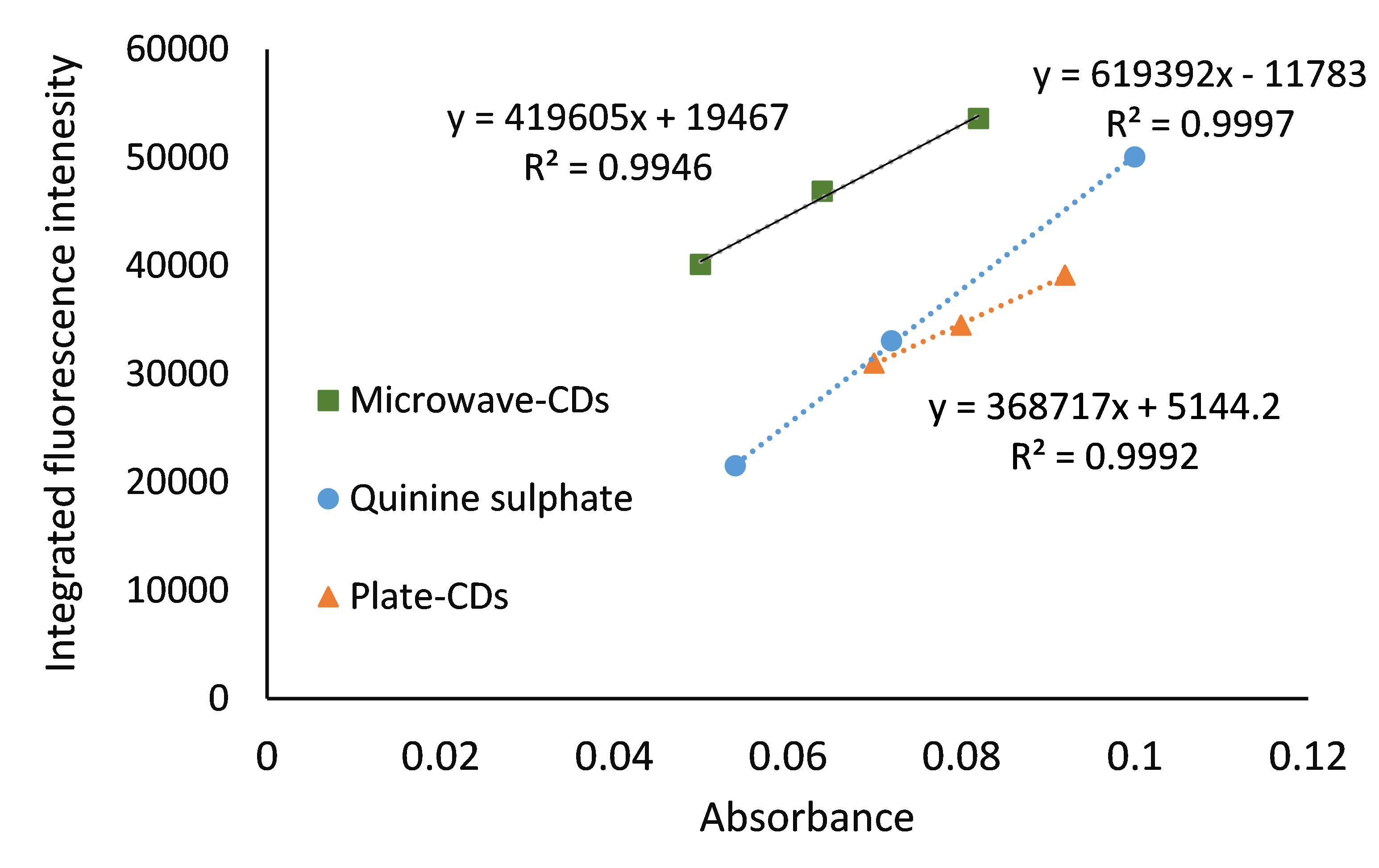


**Fig. S6**


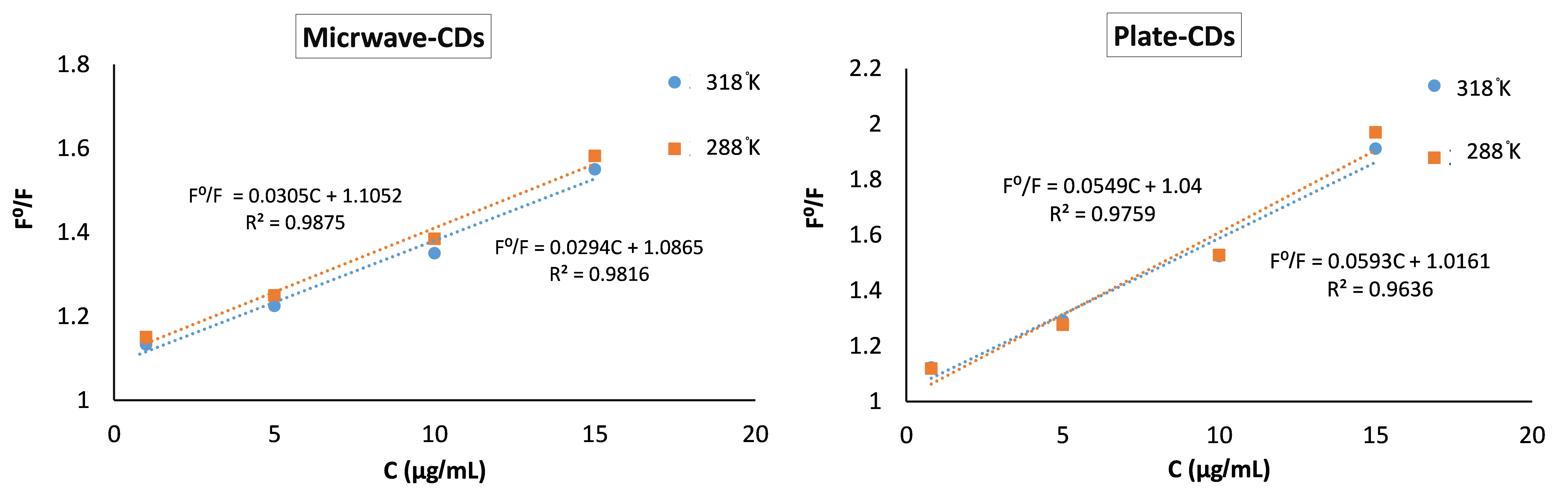


**Fig. S7**


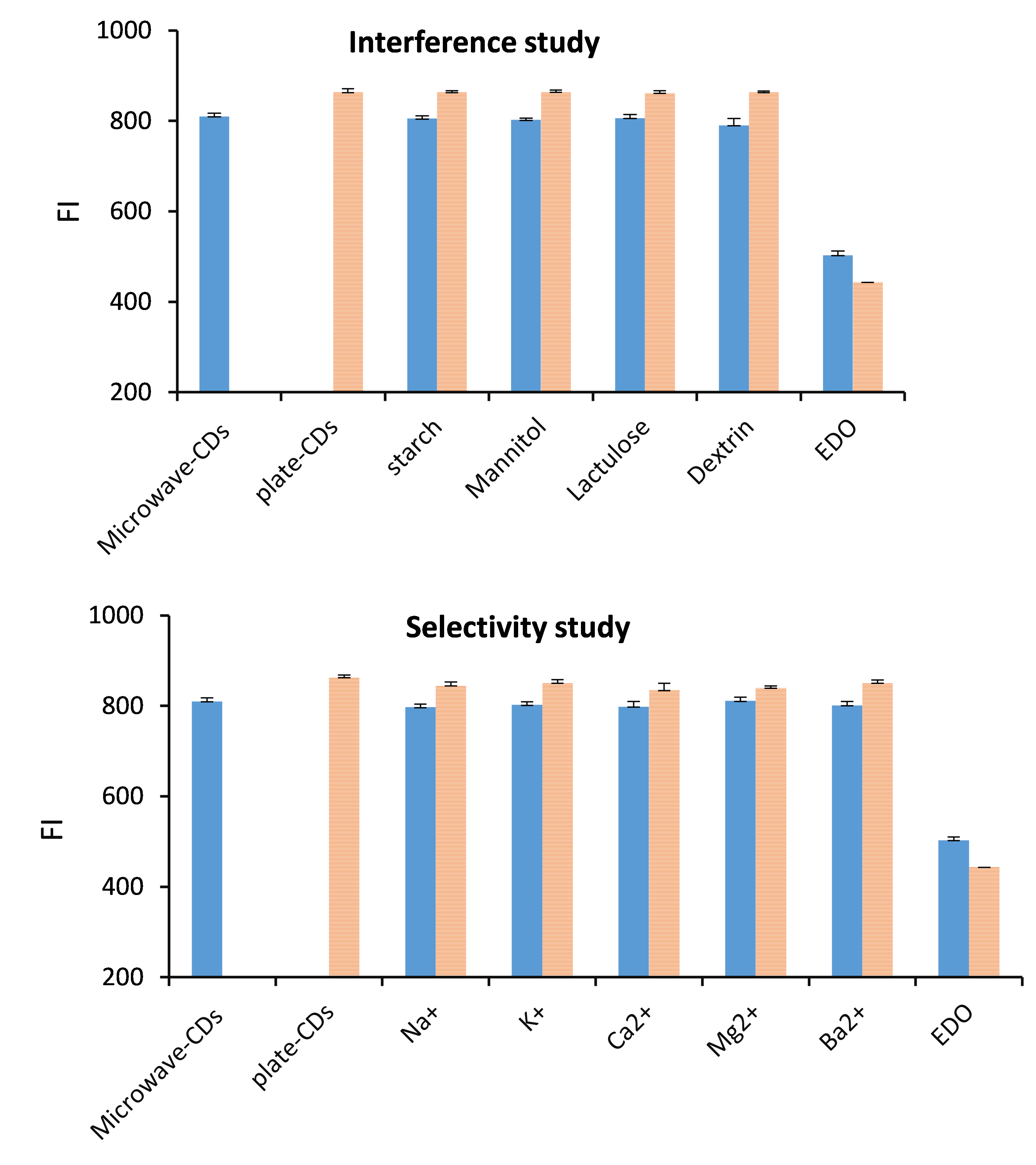


**Fig. S8**
